# Osseointegration of Anodized vs. Sandblasted Implant Surfaces in a Guided Bone Regeneration Acute Dehiscence‐Type Defect: An In Vivo Experimental Mandibular Minipig Model

**DOI:** 10.1111/clr.14369

**Published:** 2024-10-10

**Authors:** Shakeel Shahdad, Simon Rawlinson, Nahal Razaghi, Anuya Patankar, Mital Patel, Mario Roccuzzo, Thomas Gill

**Affiliations:** ^1^ Barts and the London School of Medicine and Dentistry, Institute of Dentistry Queen Mary University of London London UK; ^2^ Department of Restorative Dentistry The Royal London Dental Hospital, Barts Health NHS Trust London UK; ^3^ Private Practice Torino Italy; ^4^ Department of Maxillo‐Facial Surgery University of Torino Torino Italy; ^5^ Department of Periodontics and Oral Medicine University of Michigan Ann Arbor Michigan USA

**Keywords:** anodized, dehiscence type defects, guided bone regeneration, hydrophillic implant surface, implant crestal bone formation, implant surface, osseointegration, SLA, SLActive, TiUltra

## Abstract

**Objectives:**

This controlled preclinical study analyzed the effect of implant surface characteristics on osseointegration and crestal bone formation in a grafted dehiscence defect minipig model.

**Material and Methods:**

A standardized 3 mm × 3 mm acute‐type buccal dehiscence minipig model grafted with deproteinized bovine bone mineral and covered with a porcine collagen membrane after 2 and 8 weeks of healing was utilized. Crestal bone formation was analyzed histologically and histomorphometrically to compare three implant groups: (1) a novel, commercially available, gradient anodized (NGA) implant, to two custom‐made geometric replicas of implant “1,” (2) a superhydrophilic micro‐rough large‐grit sandblasted and acid‐etched surface, and (3) a relatively hydrophobic micro‐rough large‐grit sandblasted and acid‐etched surface.

**Results:**

At 2 and 8 weeks, there was no difference between the amount and height of newly formed bone (NBH, new bone height; BATA, bone area to total area) for any of the groups (*p* > 0.05). First bone‐to‐implant contact (fBIC) and vertical bone creep (VBC) at 2 and 8 weeks were significantly increased for Groups 2 and 3 compared to Group 1 (*p* < 0.05). At 8 weeks, osseointegration in the dehiscence (dehiscence bone‐implant‐contact; dBIC) was significantly higher for Groups 2 and 3 compared to Group 1 (*p* < 0.05).

**Conclusions:**

The amount of newly formed bone (BATA) and NBH was not influenced by surface type. However, moderately rough surfaces demonstrated significantly superior levels of osseointegration (dBIC) and coronal bone apposition (fBIC) in the dehiscence defect compared to the NGA surface at 2 and 8 weeks.

**Trial Registration:**

For this type of study, clinical trial registration is not required. This study was conducted at the Biomedical Department of Lund University (Lund, Sweden) and approved by the local Ethics Committee of the University (M‐192‐14)

## 1 Introduction

Osseointegrated dental implants are a predictable method of replacing missing teeth with high levels of success (Albrektsson et al. 2012; Jung et al. 2012; Rasperini et al. 2014; Howe, Keys, and Richards 2019). Five different implant surfaces, including sandblasted acid‐etched and anodized surfaces, are well documented, with failure rates of approximately 5% at 10 years (Wennerberg, Albrektsson, and Chrcanovic 2018). Achieving osseointegration is no longer the benchmark for dental implant surface studies, and research has focused on developing implant surfaces that induce, enhance, and maintain new bone apposition and osseointegration (Albrektsson et al. 1981; Wennerberg and Albrektsson 2009; Schwarz et al. 2007a, 2007b; Schwarz, Sager, et al. 2010; Albrektsson and Wennerberg 2019). Implant surface characteristics such as topography, chemistry, mechanical, and physical properties all interact to contribute to the biological effect of an implant. Optimization of these surface properties has permitted earlier loading protocols and aimed to maximize crestal bone levels to aid long‐term stability and aesthetics (Barfeie, Wilson, and Rees 2015).

Prosthetically driven implant placement may dictate an implant position with residual buccal bone dehiscence (Jung et al. 2017). These defects are routinely treated with guided bone regeneration (GBR) using membranes and bone substitution materials (Berglundh and Lindhe 1997; Simion et al. 1997; Zitzmann, Naef, and Schärer 1997; Hämmerle and Lang 2001; Retzepi and Donos 2010; Jung et al. 2021). Thus, implant surfaces that are osseopromoting are desired to promote bone regeneration and apposition to the crestal part of the implant. High peri‐implant bone levels and a tight coronal soft tissue barrier have been proposed as critical to the long‐term aesthetic success of implant‐supported restorations (Laurell and Lundgren 2011; Atsuta et al. 2016).

Various implant surfaces have been proposed to achieve and maintain high crestal bone levels (Laurell and Lundgren 2011; Valles et al. 2018). Differences in the surface roughness and hydrophilicity of the implant have been shown to differentially effect coronal bone growth and marginal bone loss (Schwarz et al. 2007a; Hermann et al. 2011; De Bruyn et al. 2017; Shahdad et al. 2022). From a clinical point of view, the need to regenerate dehiscence defects using GBR is commonplace in modern implantology, and its success is necessary for the long‐term biological and aesthetic success of dental implants. Previous animal studies have demonstrated that in both acute and chronic dehiscence defects, the chemically modified moderately rough sandblasted acid‐etch (modSLA) surface has a high potential to support bone formation (Schwarz et al. 2007a, 2008; Schwarz, Jung, et al. 2010; Shahdad et al. 2022). Clinical studies have corroborated this finding by demonstrating predictable long‐term outcomes for bone regeneration and stability of GBR on modSLA implants (Buser et al. 2013; Chappuis et al. 2018).

Recent theories have suggested that the marginal bone loss around rough implant surfaces might be higher compared to machined or moderately rough (surface roughness, Sa = 1–2 μm) implant surfaces (De Bruyn et al. 2017; Milleret et al. 2019; Susin et al. 2019). Therefore, a new implant concept has been developed, which is based on a novel gradient anodized (NGA) surface. This implant has a multi‐zone surface with a gradual change in topography from collar to apex. At collar level (up to 2 mm from the implant shoulder), it is minimally rough (Sa ~0.5 μm), non‐porous, with a nanostructured oxide layer. It transitions to the apex in terms of roughness (from Sa ~0.9 to 1.4 μm), and a low‐to‐high pore density (Milleret et al. 2019; Susin et al. 2019). Further, the NGA surface has demonstrated high surface energy and superhydrophilicity with a contact angle of 0°, with such properties having been associated with enhancing coronal bone apposition and osseointegration (Wennerberg et al. 2014; Milleret et al. 2019).

The benefit of such a configuration is yet to be fully explored and requires further investigation. Previous preclinical and clinical studies have demonstrated that moderately rough surfaces (surface roughness, Sa = 1–2 μm) promote osseointegration, but smooth (Sa < 0.5 μm) and minimally rough surfaces (Sa = 0.5–1 μm) may relatively favor soft tissue attachment (Wennerberg and Albrektsson 2009). Clinical studies investigating subcrestal placement of implants with a smooth (either polished or machined) to rough transition have indicated that such configurations may not yield desirable results for crestal bone maintenance (Hämmerle et al. 1996; Hartman and Cochran 2004). A recent preclinical study utilizing identical geometry demonstrated consistently higher levels of de novo bone apposition and vertical bone creep in non‐grafted acute‐dehiscence type defects for the modSLA surface compared to the NGA surface (Shahdad et al. 2022). Currently, no studies have investigated the capacity of an NGA implant that is minimally rough (Sa ~0.5 μm), non‐porous at its collar and has a nanostructured oxide layer to permit GBR in a buccal dehiscence scenario in vivo.

Therefore, this study aimed to histomorphometrically evaluate bone regeneration in NGA implants compared to modSLA and it's hydrophobic version, SLA. The relative performance was investigated using a standardized buccal acute‐type dehiscence with a GBR procedure using deproteinized bovine bone in combination with porcine collagen membrane in minipigs assessing: crestal bone regeneration and osseointegration (Rupp et al. 2006; Bosshardt, Chappuis, and Buser 2017). The study was designed to assess how the implant surface characteristics influence osseointegration and new bone formation in a guided bone regeneration dehiscence defect by comparing the healing of NGA surface implants with micro‐rough large‐grit sandblasted and acid‐etched surface implants (modSLA & SLA) after 2 and 8 weeks.

## 2 Materials and Methods

### 2.1 Study Design

This controlled preclinical study aimed to investigate the influence of surface characteristics on crestal bone formation after guided bone regeneration of a dehiscence defect and their ability to promote osseointegration. The study design has previously been reported by Shahdad et al. (2022). A mandibular minipig model using a single endpoint at 2 and 8 weeks after implantation was chosen as a test system.

To reduce the number of animals used for research, each animal received two additional study groups that were part of a different study (Shahdad et al. 2022).

This study was conducted at the Biomedical Department of Lund University (Lund, Sweden) and approved by the local Ethics Committee of the University (M‐192‐14) following the proper institutional and national guidelines for the care and use of the animals in the study. This study adhered to the ARRIVE 2.0 Guidelines and was designed by considering the 3R principle for animal research (Percie du Sert et al. 2020).

The impact of surface characteristics was investigated by comparing:

- NGA functionalized, commercially available implants (NobelActive, TiUltra NP, commercially pure titanium, 3.5ø × 8.5 mm, Nobel Biocare AG, Switzerland) (Group 1, Test group)

with two custom‐made replicas (clones) of the NGA implant geometry:

- modified with the SLActive surface, Replica of NobelActive, SLActive, Roxolid, 3.5ø × 8.5 mm, Institut Straumann AG, Switzerland (Group 2, modSLA, Control Group) and
- modified with the SLA surface, Replica of NobelActive, SLA, Roxolid, 3.5ø × 8.5 mm, Institut Straumann AG, Switzerland (Group 3, SLA, Control Group).

The characterization of Groups 2 and 3 implant geometry has been previously reported (Shahdad et al. 2022). Briefly, clone implants (Groups 2 and 3) were milled from implant blanks TiZr, followed by surface modification according to standard procedures for modSLA and SLA (Institute Straumann AG). Characterization of implant geometry using microCT (Zeiss Metrotom 1500 G3, Zeiss, Germany) for replica implants (Groups 2 and 3) was carried out before surface functionalization and has been previously reported (Shahdad et al. 2022).

A total of 15 Göttingen minipigs were included in the study. Study groups were compared by intra‐animal comparison using one type of implant (group) per animal for each study group. Implant positions were altered between animals by a rotation scheme to ensure that each implant was represented the maximum number of times at each anatomical position (left/right and mesial/distal) across the animals per healing period. Groups 1 and 3 were always located on the same hemi‐mandible, with Group 2 always placed on the contralateral side. Each study group at the different healing periods had an *n* = 7 for the 2 weeks of healing and an *n* = 8 for the 8 weeks of healing. The sample size was determined based on previous animal studies and previous experience that a minimum of *n* = 6 was necessary (Dard et al. 2016; El Chaar et al. 2021; Francisco et al. 2021; Hadaya et al. 2022).

### 2.2 Animals

Fifteen female Göttingen Minipigs (Ellegaard) of age between 20 and 24 months at the time of surgery and an average body weight of 40 kg were included in the study. The animals were housed in standard boxes in groups of three. Animals were adapted to experimental conditions by starting animal housing 1 week before intervention. Animals were fed a standard soft food diet (Special Diet Services [SDS], Witham, UK #801586). Animals were fasted overnight before surgery to prevent vomiting.

### 2.3 Surgical Procedure

All surgical procedures were performed under general anesthesia using a combination of dexmedetomidine (25–35 μg/kg i.m., Dexdomitor; Orion Pharma Animal Health) and tiletamine‐zolazepam (50–70 mg/kg i.m., Zoletil 100 Vet, Virbac) injected intramuscularly and maintained with intravenous infusion after induction with propofol (PropoVet multidose, Orion Pharma Animal Health) and fentanyl (Fentanyl B. Braun). Carprofen (4 mg/kg, s.i.d., i.m., Rimadyl vet., Orion Pharma Animal Health) was given as a pre‐emptive dose and post‐surgically up to 4 days together with buprenorphine (0.03 mg/kg, i.m., Vetergesic vet., Orion Pharma Animal Health). To reduce the dosage of the systemic anesthetic, bleeding during surgery, and to alleviate post‐surgical pain, local anesthesia was provided intraoperatively by infiltrative injection of 1.8 mL of Xylocaine (Xylocaine, Dental adrenalin, 20 mg/mL and 12.5 μg/mL; Astra AB) per hemi‐mandible. Antibiotic prophylaxis was administered using benzylpenicillin prokain‐dihydrostreptomycin (25 mg/kg + 20 mg/kg, s.i.d., i.m., Streptocillin vet., Boehringer Ingelheim Vetmedica). Animals were intubated and breathing withheld by a ventilator. Vital parameters were monitored continuously (pulse oximetry, rectal temperature, blood pressure, CO_2_).

### 2.4 Tooth Extraction

Three contralateral mandibular premolars (P2–P4) and first mandibular molars (M1) were carefully extracted without raising a flap using a minimally invasive surgical approach.

### 2.5 Implant Osteotomy and Implant Placement

**FIGURE 1:**
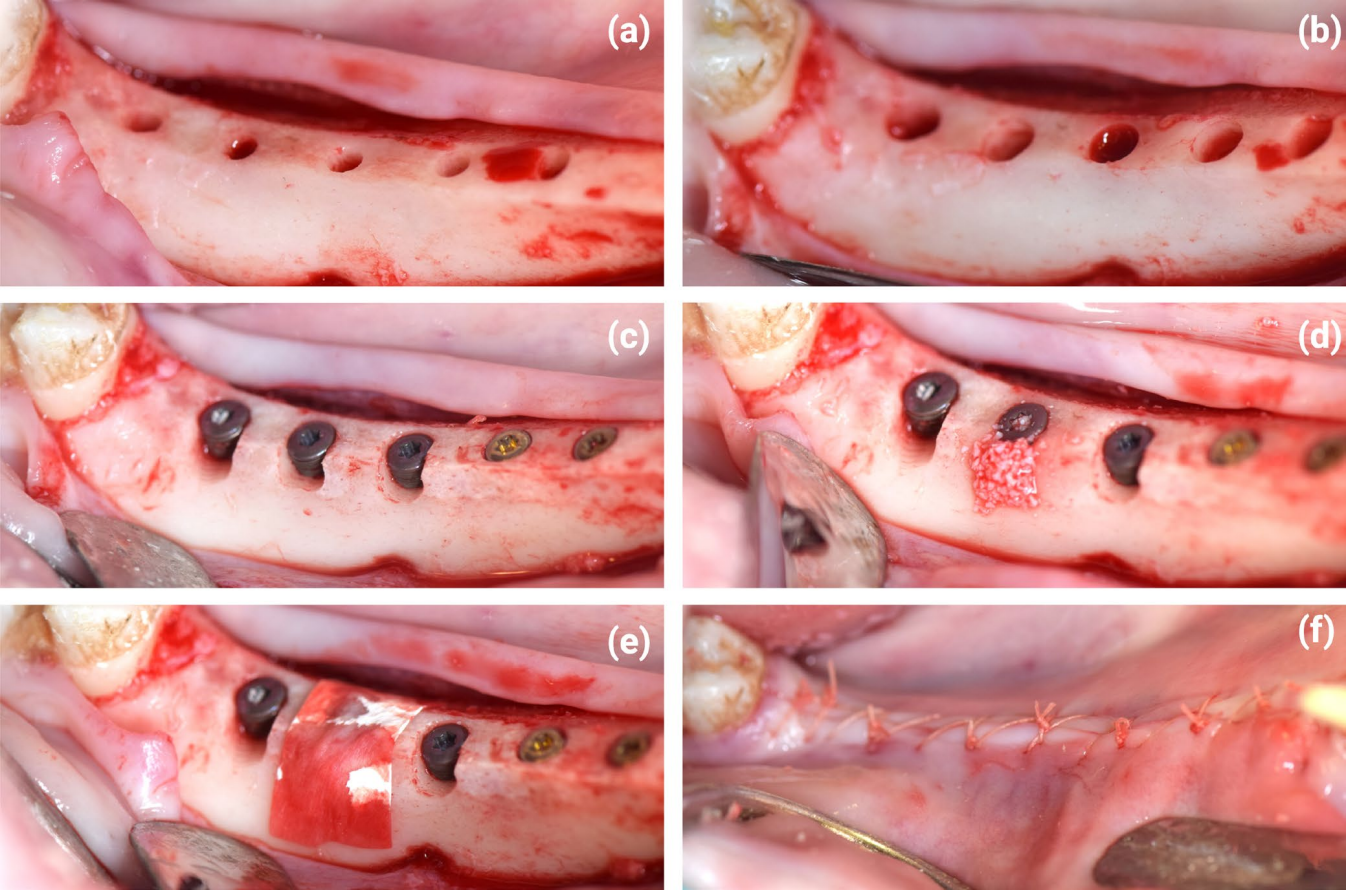
Illustration of surgical procedures. (a) implant osteotomy preparation starting from a flattened mandibular alveolar ridge. (b) creation of osteotomy. (c) implant placement with standardized acute‐type buccal dehiscence type defect (3 × 3 × 3 mm). (d) grafting of dehiscence defect with deproteinized bovine bone mineral. (e) coverage with a collagen membrane. (f) primary wound closure.

Implants were placed 20 weeks' post‐extraction. Mandibular alveolar ridges were exposed by elevation of a mucoperiosteal flap after midcrestal incision and flattened using a cylindrical cutting bur under saline irrigation. Implant positions for the test and control implants were rotated between left/right and the P3, P4, and M1 positions (Figure 1).

Implant osteotomies were prepared according to the manufacturer's instructions using the corresponding drills and drill sequences. In brief, osteotomies for all the groups were prepared as per the manufacturer's guidelines for hard bone, using the sequence: Ø2.0 → Ø2.4/2.8 → Ø2.8/3.2 → Tap Drill (Nobel Twist and Step drills, Nobel Biocare AG). Following osteotomy preparation, buccal dehiscence‐type defects (3 × 3 × 3 mm) were created with a Lindemann drill as previously described (Shahdad et al. 2022).

Implants were placed at 1 mm subcrestal to the lingual crest using a motorized handpiece and a custom‐made torque ratchet (Institut Straumann AG). Primary implant stabilities were assessed in terms of maximum insertion torques (max IT). Implants were subsequently equipped with closure screws. The standardized 3 mm acute‐type buccal dehiscence was grafted with deproteinized bovine bone mineral (DBBM‐Bio‐Oss, Geistlich) hydrated in saline and covered with a porcine collagen membrane (Bio‐Gide, Geistlich), followed by primary wound closure (Vicryl 4.0, Ethicon) for submerged healing. Antibiotic cover and analgesia, as described above, were administered for 7 days post‐surgery (Streptocilin vet, Boehringer Ingelheim, 3–4 mL/pig i.m.).

### 2.6 Termination

Animals were sacrificed by intra‐cardiac injection of a 20% solution of pentobarbital (Pentobarbitalnatrium, Apoteket AB; 60 mg/mL). Block sections of the implant sites were prepared with an oscillating autopsy saw under perseveration of the soft tissues and fixed in formalin (4% formaldehyde solution) for at least 2 weeks before histological processing.

### 2.7 Histological Processing

Formalin‐treated block sections were dehydrated using ascending grades of alcohol and xylene and, subsequently, infiltrated and embedded in methyl methacrylate (MMA, Sigma Aldrich; polymerized by Perkadox 16, Nouryon) for non‐decalcified sectioning. Block sections were then cut in a buccolingual direction to sections of 500 μm (Exakts, Apparatebau, Norderstedt, Germany) (1 central section per implant) and ground to a final thickness of 30–50 μm. Sections were stained with paragon (toluidine blue and basic fuchsin) for microscopic evaluation.

### 2.8 Quantitative Histomorphometry

Histomorphometric parameters, as previously described by Shahdad et al. (2022), were evaluated on central buccolingual sections of the implant and exclusively buccal aspects of the implant where the GBR occurred.

The primary outcome of this study was bone‐to‐implant contact in the dehiscence defect (dBIC). The histomorphometric parameter directly associated with this outcome was as follows.

- The percentage of bone‐to‐implant contact in the coronal 3 mm of the implant, where the acute‐dehiscence defect was grafted.

Secondary outcomes related to the capacity of the individual implant surfaces to promote osseointegration and bone apposition included the following:

- First bone‐to‐implant contact (fBIC) is calculated by the distance between the implant shoulder and the most coronal aspect of the bone in direct contact with the implant.
- Bone area to total area (BATA) is the ratio between the area occupied by newly formed bone and the total defect area.
- Vertical bone creep (VBC) is defined by the height of newly formed bone within the dehiscence defect area in direct contact with the implant measured from the bottom of the defect.
- New bone height (NBH) is defined by the maximum crestal height of the newly formed bone crest in the defect measured from the lowest point of the original defect.

### 2.9 Statistical Evaluation

Values for measured parameters were summarized as means, standard deviations, medians, and interquartile ranges. Test and control implants of individual groups were compared using the Friedman test to assess maximum insertion torque at 2 and 8 weeks separately. Since neither *p*‐value reached the significance threshold, a post hoc analysis (Dunn test) was not performed to make pairwise comparisons.

Adjusted histomorphometric parameters, 95% confidence intervals (CI), and associations to the different test items under consideration of the factor mandible side and position in the mandible were calculated individually for the 2 and 8‐week time points using mixed linear regression models, with the factor “animal” modeled as a random effect, and adjusted for multiple comparison using the Dunnett‐Hsu method with a significance level of α < 0.05. The assumptions of the model were checked to ensure they were not violated.

The adjusted means and the 95% confidence intervals extracted from the models are reported throughout the manuscript. The software SAS version 9.4 (2016, SAS Institute Inc., Cary) was used for the analysis. The complete set of results from the statistical models is provided as part of supplementary information.

## 3 Results

### 3.1 Implant Geometry Comparisons Between NGA Test Implants and Geometric Replicas and Surface Characterization

To ensure comparable geometries, specifically at the coronal aspect of the implant, where the GBR procedure was undertaken, the three‐dimensional implant geometries of all three implants were compared by microCT (Shahdad et al. 2022). Both Group 2 and 3 replicas were created from the same batch and only differed in their subsequent surface processing. Therefore, no difference at the microCT level was observed between them.

Scanning electron microscopy (SEM) images and surface roughness measurements of Group 1 (NGA) revealed four zones with changing surface topographies and different Sa values (Shahdad et al. 2022). Specifically, Sa values measured 0.680 ± 0.079 μm, at the first transition zone, 3–4 mm from the coronal platform. SEM images of Groups 2 and 3 revealed a homogenous surface topography with the Sa values of Groups 2 and 3 measuring 1.206 ± 0.078 μm and 1.168 ± 0.061 μm, respectively.

Groups 1 and 2 demonstrated superhydrophilic characteristics, with advancing contact angles of 0 ± 0°. Group 3 displayed a comparatively less hydrophilic contact angle of 99.5 ± 8.6°.

#### 3.1.1 Animal Response to Implantation and Primary Stability Assessments

All animals recovered from surgery in a predictable manner and without any intra‐or post‐surgical complications. One of the animals sacrificed at the 2‐week time point displayed an osteoporotic phenotype as identified during histological processing, characterized by a very thin amount of crestal mandibular bone and a correspondingly large medullary cavity. The resultant histometric measurements were found to be outliers and were thus excluded from the analysis, resulting in 6 and 8 implants per test group for the 2‐ and 8‐weeks' time points, respectively.

The Friedman test found no difference for maximum insertion torque between the 3 groups at 2 or 8 weeks (2 week: *p* = 0.692, 8 week: *p* = 0.692). The average maximum insertion torque was 42.67 Ncm (95% CI: 31.90–53.44), 44.00 Ncm (95% CI: 29.21–58.79), and 33.47 Ncm (95% CI: 25.14–41.79) for Groups 1, 2, and 3, respectively.

**FIGURE 2:**
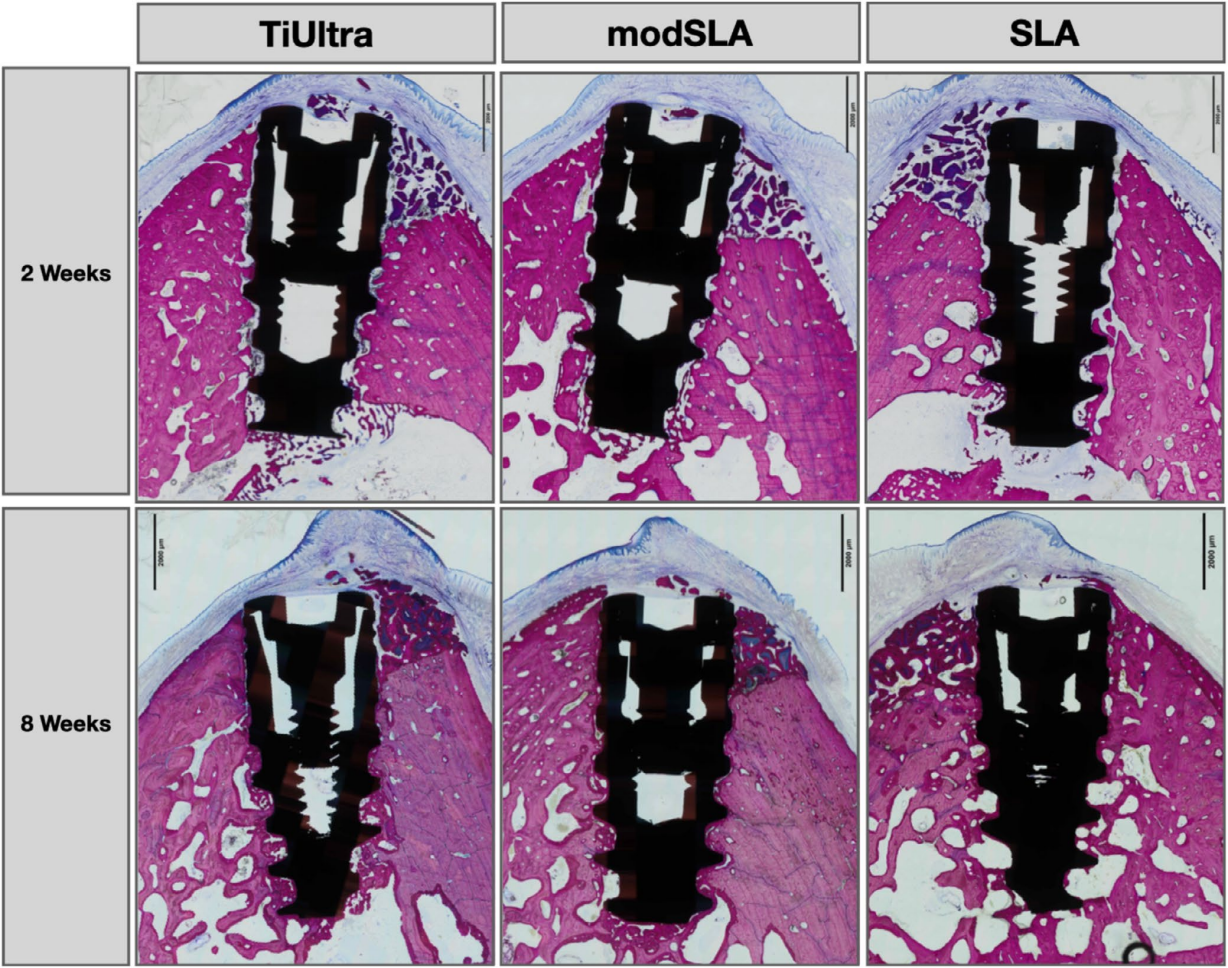
Representative micrographs of histological cross‐sections comparing the healing pattern of grafted dehiscence defects around NGA test implants (1), moderately rough modSLA geometrical clone implants (2), and moderately rough SLA geometrical clone implants (3) after 2 weeks (upper row) and 8 weeks of healing (lower row). Sections were stained with paragon (toluidine blue and basic fuchsin) for microscopic evaluation.

Figure 2 illustrates the histological cross‐sections between study groups for the buccal dehiscence defects grafted with deproteinized bovine bone at 2 and 8 weeks of healing. All implants healed well and osseointegrated without any signs of fibrous encapsulation. Qualitative differences regarding crestal bone apposition and osseointegration in the dehiscence were apparent, with Groups 2 and 3 displaying a similar increased crestal height and amount of newly formed bone compared to Group 1.

These surface‐type associated differences were further analyzed by histology, comparing the detailed healing patterns for the groups at higher magnification.

Healing for all three groups after 2 weeks was characterized by the presence of similar volumes of bone graft material in the dehiscence defect with a provisional matrix and new bone forming from the apical margin of the defect. After 8 weeks, the dehiscence defects displayed the formation of mature lamellar bone from the apical margin coronally around the bone graft material, resulting in similar amounts of new bone height and volume around the bone graft.

**FIGURE 3:**
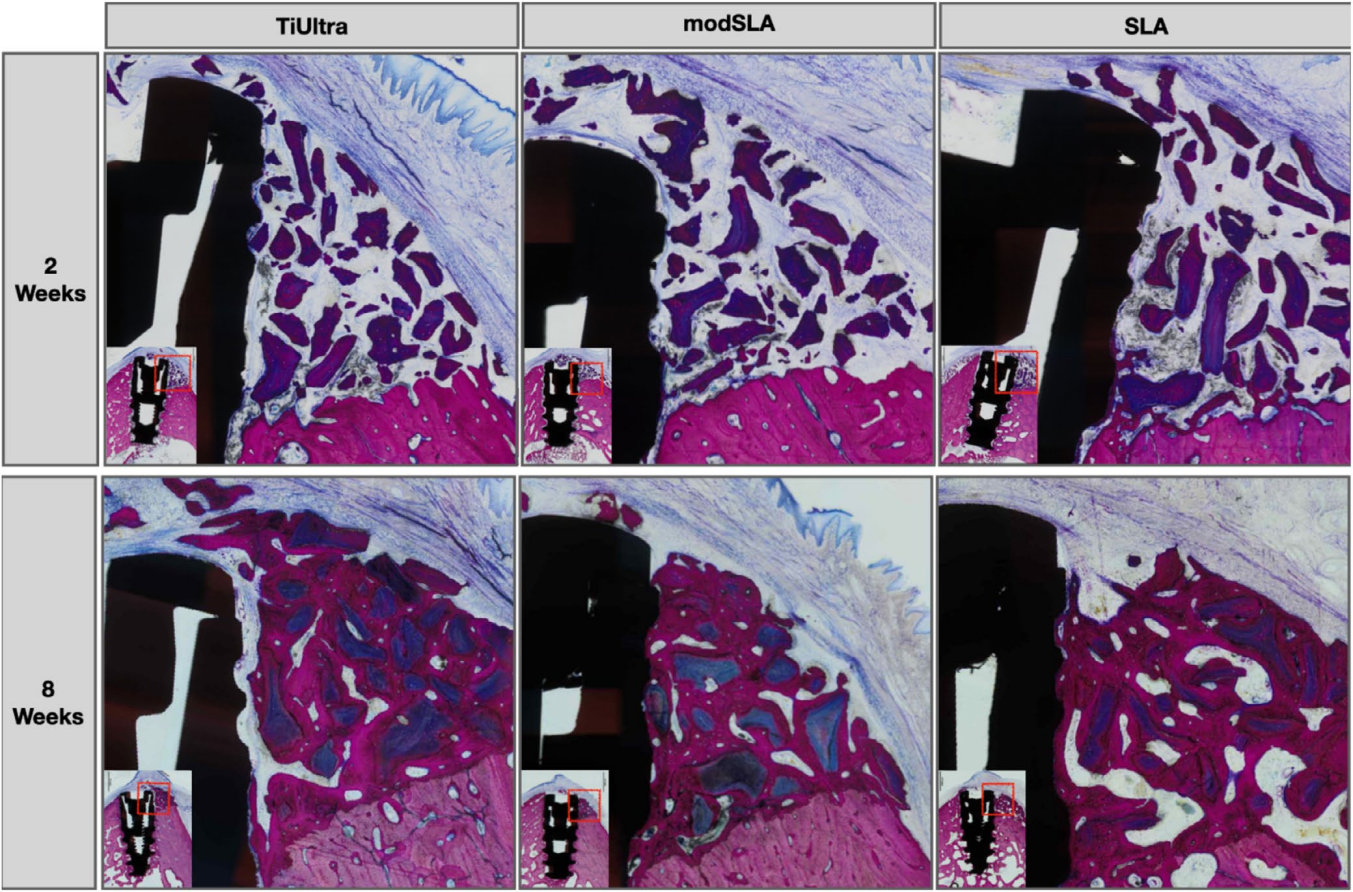
Representative micrographs at higher magnification of histological cross‐sections comparing the healing pattern of grafted dehiscence defects around NGA test implants (1), moderately rough modSLA geometrical clone implants (2), and moderately rough SLA geometrical clone implants (3) after 2 weeks (upper row) and 8 weeks of healing (lower row). The 2 and 8 week SLA micrographs have been mirrored to allow for easier viewing. Sections were stained with paragon (toluidine blue and basic fuchsin) for microscopic evaluation.

As evidenced by the histological micrographs in Figure 3, distinct differences in the healing patterns for Group 1 compared to Groups 2 and 3 were identified at both healing points. After 2 weeks, differences were related to the degree of mineralization of newly formed bone and the quantity of direct bone apposition to the implant surface (Figure 3). At the 8‐week time point, differences were mainly related to direct bone apposition and the height to which this contacted the implant (Figure 3).

Specifically, after 2 weeks, the healing for Group 1 was characterized by limited new trabecular bone of relatively low mineralization towards the apical aspect of the defect. In contrast, Groups 2 and 3 demonstrated comparatively higher coronal bone apposition of newly formed bone in direct contact with the implant surface.

After 8 weeks of healing, the morphology of crestal bone associated with Group 1 displayed a wedge‐shaped defect‐like morphology around the implant surface. This presented as a detectable slit‐like void between the newly formed bone and the implant surface. As seen in Figure 3, despite new bone formation surrounding the bone graft particles and similar in maturity and height to Groups 2 and 3, Group 1 demonstrated more apically positioned direct contact of newly formed bone with the implant. Groups 2 and 3 by contrast demonstrated mature lamellar bone more coronally in direct contact with the implant surface.

#### 3.1.2 Histomorphometry

To investigate the height and amount of newly formed bone in the dehiscence defect as a function of group, the histomorphometric outcomes of NBH and BATA were compared after 2 and 8 weeks of healing. To determine the effect of the groups on osseointegration, the coronal extent to which new bone formed and the extent of direct bone apposition to the implant surface VBC, fBIC, and dBIC were assessed.

**FIGURE 4:**
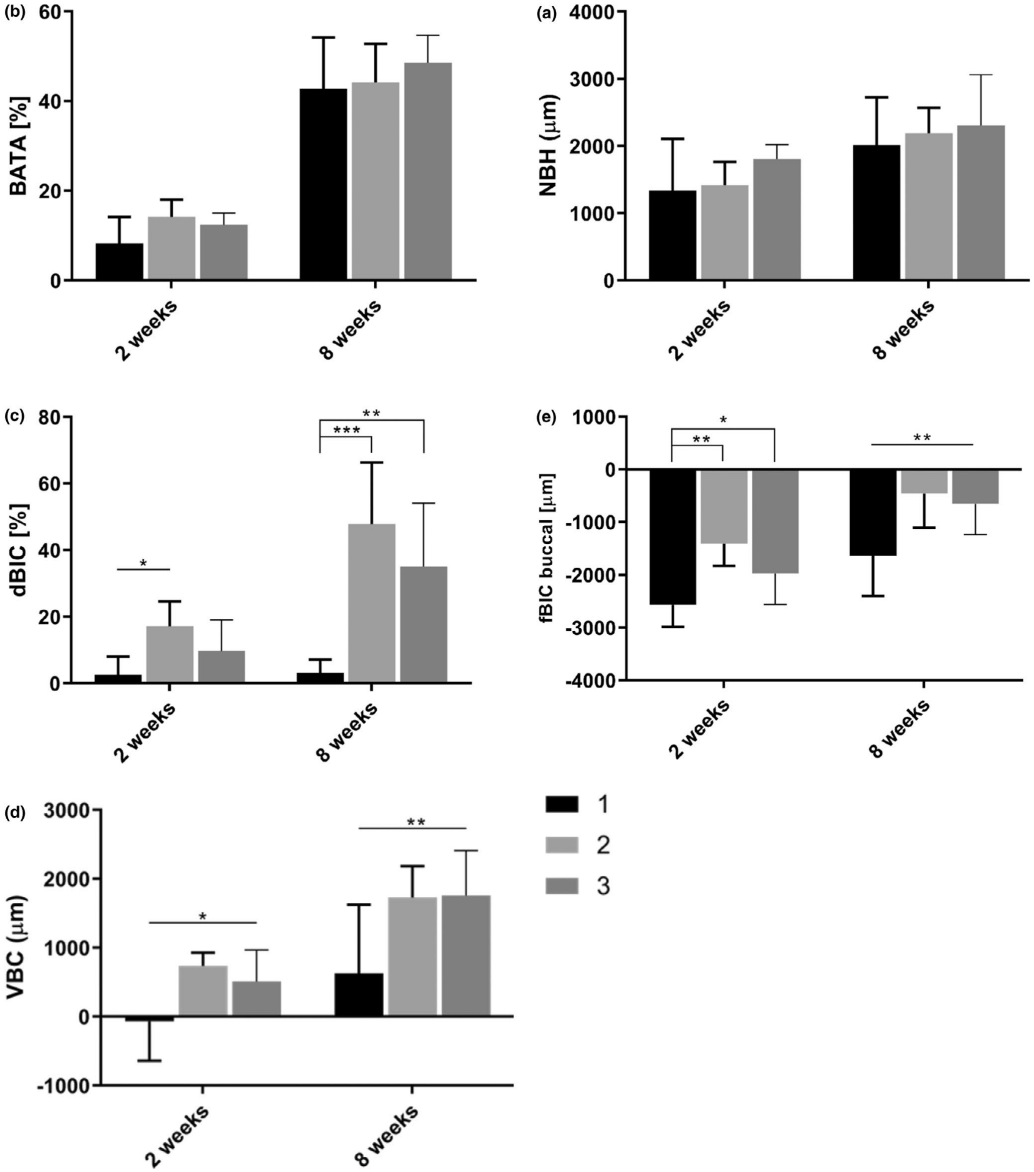
Comparison of histomorphometric parameters between different implant groups after 2 and 8 weeks of healing: (a) NBH, new crestal bone height; (b) BATA, ratio of bone area to total area in the defect; (c) dBIC, bone‐to‐implant contact in the dehiscence defect area; (d) VBC, vertical bone creep; (e) fBIC, first bone‐to‐implant contact. 1—Group 1 NGA test implants, 2—Group 2 moderately rough modSLA geometrical clone implants and 3—Group 3 moderately rough SLA geometrical clone implants. Individual values represent adjusted mean values by mixed linear regression. Error bars designate the 95% confidence intervals. Levels of significance as adjusted according to Dunnett‐Hsu: *p ≤ 0.05, **p ≤ 0.01, ***p ≤ 0.001.

The descriptive statistics (Table 1) and statistical comparisons of Tables 2 and 3 indicate that differences seen at the 2‐week point became more pronounced at 8 weeks (Figure 4). (For the full regression model output see also Tables S1 and S2).

**TABLE 1:** Descriptive statistics of histometric parameters.

Time point	Outcome	Parameter	Test item (with GBR)		
			Group 1	Group 2	Group 3
2 weeks	NBH [mm]	*n*	6	6	6
		Mean ± SD	1.34 ± 0.77	1.42 ± 0.35	1.80 ± 0.22
		Median (IQR)	1.413 (1.003–1.95)	1.54 (1.24–1.66)	1.84 (1.59–1.94)
		Median (range)	1.41 (0.04–2.19)	1.54 (0.82–1.72)	1.84 (1.52–2.10)
	VBC [mm]	*n*	6	6	6
		Mean ± SD	−0.07 ± 0.57	0.74 ± 0.19	0.51 ± 0.46
		Median (IQR)	−0.18 (−0.49 to 0.04)	0.76 (0.57–0.84)	0.45 (0.30–1.00)
		Median (range)	−0.18 (−0.61 to 1.00)	0.76 (0.47–1.01)	0.45 (−0.18 to 1.05)
	fBIC: buccal first bone‐to‐implant contact [mm]	*n*	6	6	6
		Mean ± SD	−2.57 ± 0.42	−1.41 ± 0.42	−1.97 ± 0.59
		Median (IQR)	−2.40 (−2.74 to −2.29)	−1.43 (−1.77 to −0.991)	−1.90 (−2.5 to −1.41)
		Median (range)	−2.40 (−0.33 to −2.24)	−1.43 (−1.89 to −0.93)	−1.90 (−2.74 to −1.36)
	dBIC: Bone‐to‐implant contact [%]	*n*	6	6	6
		Mean ± SD	2.55 ± 5.49	17.12 ± 7.53	9.76 ± 9.29
		Median (IQR)	0 (0–1.60)	17.96 (16.79–21.92)	6.59 (2.79–20.71)
		Median (range)	0 (0–13.68)	17.96 (3.11–24.98)	6.59 (0–21.86)
	(BA/TA) Bone area/total area [%]	*n*	6	6	6
		Mean ± SD	8.30 ± 5.91	14.21 ± 3.81397345	12.36 ± 2.66
		Median (IQR)	9.40 (2.22–13.43)	12.94 (12.62–14.73)	11.83 (11.16–14.86)
		Median (range)	9.40 (1.49–13.88)	12.94 (10.491–21.48)	11.83 (8.60–15.87)
8 weeks	NBH [mm]	*n*	8	8	8
		Mean ± SD	2.02 ± 0.71	2.19 ± 0.38	2.31 ± 0.76
		Median (IQR)	1.88 (1.46–2.74)	2.30 (2.15–2.40)	2.25 (1.94–2.85)
		Median (range)	1.88 (1.06–2.92)	2.30 (1.32–2.51)	2.25 (0.99–0.34)
	VBC [mm]	*n*	8	8	8
		Mean ± SD	0.63 ± 1.00	1.73 ± 0.46	1.76 ± 0.65
		Median (IQR)	0.13 (−0.10 to 1.47)	1.86 (1.53–2.02)	1.87 (1.30–2.15)
		Median (range)	0.13 (−0.34 to 2.36)	1.86 (0.81–2.19)	1.87 (0.72–2.70)
	fBIC: buccal first bone‐to‐implant contact [mm]	*n*	8	8	8
		Mean ± SD	−1.63 ± 0.76	−0.46 ± 0.64	−0.65 ± 0.58
		Median (IQR)	−1.76 (−2.23 to −0.91)	−0.26 (−0.45 to −0.16)	−0.35 (−1.08 to −0.30)
		Median (range)	−1.76 (−2.66 to −0.60)	−0.26 (−2.00 to 0.05)	−0.35 (−1.64 to −0.11)
	dBIC: Bone‐to‐implant contact [%]	*n*	8	8	8
		Mean ± SD	3.20 ± 3.89	47.82 ± 18.49	35.12 ± 18.96
		Median (IQR)	1.22 (0.22–6.21)	50.57 (40.40–60.13)	41.53 (19.92–50.27)
		Median (range)	1.22 (0–10.26)	50.57 (10.15–70.18)	41.53 (2.65–54.84)
	(BA/TA) Bone area/total area [%]	*n*	8	8	8
		Mean ± SD	42.80 ± 11.43	44.22 ± 8.56	48.58 ± 6.15
		Median (IQR)	48.59 (33.90–50.05)	41.21 (38.35–49.27)	51.18 (46.29–52.32)
		Median (range)	48.59 (22.44–54.86)	41.21 (35.29–60.77)	51.18 (35.87–53.22)

Abbreviations: BATA, bone area/total area; dBIC, dehiscence bone‐to‐implant contact; fBIC, first bone‐to‐implant contact; IQR, interquartile range (from Q1 to Q3); NBH, new bone height; range, minimum to maximum; SD, standard deviation; VBC, vertical bone creep.

**TABLE 2:** Adjusted association between histomorphometric outcomes after 2 weeks of healing and implant group derived from multivariable mixed linear regression models (CI, confidence interval; SE, standard error).

Outcome	Facture	Value	Regression parameters			Adjusted parameters for multiple comparisons			
			Estimate	SE	*p*	Adjusted mean	95% CI for the adjusted mean	*p* ^*^ Ref. level is group 1	*p* ^*^ Ref. level is group 2
NBH[mm]	Group	1	−0.58	0.31	0.102	1.24	0.73–1.75	*Ref*.	0.802
		2	−0.41	0.32	0.246	1.41	0.89–1.93	0.808	*Ref*.
		3	0			1.82	1.30–2.33	0.174	0.388
VBC[mm]	Group	1	−0.63	0.28	0.059	−0.14	−0.94	*Ref*.	0.034
		2	0.22	0.29	0.484	0.71	0.23–0.12	0.034	*Ref*.
		3	0			0.49	0.02–0.97	0.104	0.691
fBIC[mm]	Group	1	−0.71	0.25	0.026	−2.62	−1.02	*Ref*.	0.005
		2	0.42	0.26	0.154	−1.49	−1.03	0.005	*Ref*.
		3	0			−1.91	−1.01	0.047	0.253
dBIC[%]	Group	1	−7.90	4.85	0.147	2.09	−16.32	*Ref*.	0.0419
		2	6.00	5.07	0.276	15.99	7.75–24.24	0.043	*Ref*.
		3	0			9.99	1.85–18.14	0.247	0.430
BATA[%]	Group	1	−3.65	2.34	0.162	8.63	3.92–13.33	*Ref*.	0.082
		2	1.87	2.45	0.47	14.15	9.39–18.91	0.084	*Ref*.
		3	0			12.28	7.59–16.96	0.271	0.676

*Note:* For full regression model output, see Table S1. *p*‐values were adjusted for multiple comparisons using the Dunnett‐Hsu method. Ref.: Reference level for the comparison for different values of one individual factor.

Abbreviations: BATA, ratio of bone area to total area; dBIC, bone‐to‐implant contact in the defect area; fBIC, First bone‐to‐implant contact; NBH, new bone height; VBC, vertical bone creep.

^*^ *p* ≤ 0.05.

**TABLE 3:** Adjusted association between histomorphometric outcomes after 8 weeks of healing and implant group derived from multivariable mixed linear regression models (CI, confidence interval; SE, standard error).

Outcome	Facture	Value	Regression parameters			Adjusted parameters for multiple comparisons			
			Estimate	SE	*p*	Adjusted mean	95% CI for the adjusted mean	*p* ^*^ Ref. level is group 1	*p* ^*^ Ref. level is group 2
NBH[mm]	Group	1	−0.31	0.29	0.309	2.02	1.50–2.54	*Ref*.	0.771
		2	−0.13	0.29	0.653	2.20	1.68–2.72	0.771	*Ref*.
		3	0			2.33	1.81–2.85	0.483	0.859
VBC[mm]	Group	1	−1.16	0.31	0.004	0.64	0.14–1.14	*Ref*.	0.009
		2	−0.06	0.31	0.854	1.74	1.24–2.24	0.009	*Ref*.
		3	0			1.80	1.30–2.30	0.007	0.975
fBIC[mm]	Group	1	−0.98	0.30	0.008	−1.61	−0.95	*Ref*.	0.005
		2	0.19	0.30	0.544	−0.44	−0.95	0.005	*Ref*.
		3	0			−0.63	−0.95	0.014	0.762
dBIC[%]	Group	1	−31.85	7.22	0.001	3.59	−22.41	*Ref*.	0.0001
		2	12.77	7.22	0.105	48.21	37.01–59.42	0.0001	*Ref*.
		3	0			35.44	24.24–46.64	0.002	0.179
BATA[%]	Group	1	−6.24	4.77	0.217	42.48	35.08–49.88	*Ref*.	0.937
		2	−4.83	4.77	0.333	43.89	36.50–51.29	0.937	*Ref*.
		3	0			48.72	41.32–56.12	0.353	0.516

*Note:* For full regression model output, see Table S2. *p*‐values were adjusted for multiple comparisons using the Dunnett‐Hsu method. Ref.: Reference level for the comparison for different values of one individual factor.

Abbreviations: BATA, ratio of bone area to total area; dBIC, bone‐to‐implant contact in the defect area; fBIC, first bone‐to‐implant contact; NBH, new bone height; VBC, vertical bone creep.

^*^ *p* ≤ 0.05.

Crestal bone height after both 2 and 8 weeks, as assessed by NBH, showed no evidence of a difference (*p* > 0.05) between any of the groups (2 week: Group 1 vs. 2 *p* = 0.808, Group 1 vs. 2 *p* = 0.174, Group 2 vs. 3 *p* = 0.388); (8 week: Group 1 vs. 2 *p* = 0.771, Group 1 vs. 3 *p* = 0.483, Group 2 vs. 3 *p* = 0.859) (Tables 1 and 2, Figure 4). All groups, on average, increased in NBH between the two time points.

The amount of newly formed crestal bone as evaluated in terms of BATA, similarly, showed no evidence of any difference (*p* > 0.05) between the groups at either timepoint (2 week: Group 1 vs. 2 *p* = 0.084, Group 1 vs. 3 *p* = 0.271, Group 2 vs. 3 *p* = 0.676); (8 week: Group 1 vs. 2 *p* = 0.937, Group 1 vs. 3 *p* = 0.353, Group 2 vs. 3 *p* = 0.516) (Tables 1 and 2).

Bone apposition and osseointegration as assessed in terms of VBC and fBIC at 2w and dBIC, VBC, and fBIC at the 8‐week time point were consistently and statistically significantly higher for Groups 2 and 3, compared to Group 1.

Specifically, at 2 weeks, dBIC was significantly higher (*p* = 0.043) for Group 2 (mean = 15.99%, 95% CI: 7.75%–24.34%) compared to Group 1 (mean = 2.09%, 95% CI: −16.32%). There was no difference observed between Groups 1 and 3 (*p* = 0.247, Group 3 mean = 9.99%, 95% CI: 1.85%–18.14%). At 8 weeks, both Group 2 (mean = 48.21%, 95% CI: 37.01%–59.42%) and Group 3 (mean = 35.44%, 95% CI: 24.24%–46.64%) demonstrated significantly higher osseointegration in terms of dBIC, compared to Group 1 (mean = 3.59%, 95% CI: −22.41%) (Group 1 vs. Group 2 *p* < 0.001, Group 1 vs. Group 3 *p* = 0.002, Group 2 vs. Group 3 *p* = 0.179).

After both 2 and 8 weeks, Group 2 showed significantly higher crestal bone formation at the implant surface in terms of VBC compared to Group 1 (Group 1 vs. 2, 2 week *p* = 0.034, 8 week *p* = 0.009) (Tables 1 and 2). Further, Group 3 at 8 weeks demonstrated significantly higher VBC compared to Group 1 (Group 1 vs. 3 *p* = 0.007). At 8 weeks, Group 3 (mean = 1.80 mm, 95% CI: 1.30–2.30 mm) and Group 2 (mean = 1.74 mm, 95% CI: 1.24–2.24 mm) on average demonstrated the highest values for VBC, followed by Group 1 (mean = 0.64 mm, 95% CI: 0.14–1.14 mm).

At 2 and 8 weeks, crestal bone apposition as measured by fBIC was significantly more coronal in Groups 2 and 3 compared to Group 1 (2 week: Group 1 vs. 2 *p* = 0.005, Group 1 vs. *p* = 0.047, 8 week: Group 1 vs. 2 *p* = 0.005, Group 1 vs. 3 *p* = 0.014) (Tables 1 and 2). At 8 weeks, Group 2 averaged the most coronal bone apposition (mean = −0.44 mm, 95% CI: −0.95 mm), followed by Group 3 (mean = −0.63 mm, 95% CI: −954 mm) and Group 1 (mean = −1.61 mm, 95% CI: −0.95 mm).

## 4 Discussion

This study investigated the influence of implant surface properties on osseointegration using guided bone regeneration in a standardized buccal acute‐type dehiscence model. The impact of implant surface properties was examined by comparing the superhydrophilic novel gradient anodized surface (NGA, Group 1) with cloned superhydrophilic moderately rough large‐grit sandblasted and acid‐etched surface (modSLA, Group 2) and relatively hydrophobic moderately rough large‐grit sandblasted and acid‐etched surface (SLA, Group 3).

From the comparison of the study groups, the following main observations were made: (A) Although NBH at 2 and 8 weeks was greater for Groups 2 and 3 compared to Group 1, these differences were not statistically different. Therefore, there was no evidence to support differences in surface properties affected by NBH. However, the relationship of this regenerated bone was distinctly different for Group 1, with significantly less bone‐to‐implant contact in the dehiscence defect. (B) Similarly, for BATA, there was no statistical evidence to suggest a difference between groups at either timepoint. All three groups saw increases in BATA from the 2‐week to the 8‐week timepoint. Again, the surface properties did not significantly influence the height and volume of newly formed bone within the dehiscence defect. (C) At 2 weeks Group 2 demonstrated significantly higher dBIC percentages compared to Group 1. This increased in significance at the 8‐week timepoint. Further, Group 3 demonstrated a significantly higher dBIC percentage at 8 weeks. Thus, suggesting differences may be associated with surface properties. (D) All remaining defect‐related variables (VBC, and fBIC) were superior for implant Groups 2 and 3 at 8 weeks of healing, which also suggests that differences may be associated with surface properties.

Histomorphometric measurements and qualitative assessment for NBH and BATA demonstrated no difference between the groups, confirming that the GBR with deproteinized bovine bone covered with porcine collagen membrane predictably formed new bone in the defect around the three implant surfaces investigated in this study. However, when assessing dBIC and fBIC, the pronounced absence of direct apposition of new bone at the coronal aspect of Group 1 (NGA) indicated that the surface properties directly influenced crestal bone formation. Thus, supporting our hypothesis that NGA surface implants demonstrate significantly lower levels of bone‐to‐implant contact in the dehiscence, as measured by dBIC, after 8 weeks of healing compared to micro‐rough large‐grit sandblasted and acid‐etched surface implants (modSLA & SLA). These results correlate with those reported by us previously (Shahdad et al. 2022) in a spontaneous healing model, where the same size defects were utilized but without a bone graft. In that study too, the NGA surface formed a wedge‐like gap at the coronal aspect of the dehiscence defect that transitioned into a thin, non‐mineralized slit‐like gap interposed between the implant surface and newly formed lamellar bone. The clinical implications and stability of such a presentation are unclear and may potentially make them more susceptible to future bone loss and soft tissue recession.

Surface characteristics like topography, wettability, and coatings contribute to the biological processes during osseointegration through a range of mechanisms, such as mediating the direct interaction of host osteoblasts in bone formation, enhancing blood clot stabilization, or modifying the host‐inflammatory response (Schwarz et al. 2007b; Hotchkiss et al. 2017; Lotz et al. 2017). Groups 1 and 2 displayed superhydrophilic properties. The NGA surface (Group 1) achieves this through a protective salt layer (Rupp et al. 2006; Lüers, Laub, and Jennissen 2016; Milleret et al. 2019). In contrast, the moderately rough surface of Group 2 (modSLA) is maintained through wet storage. Group 3 (SLA) demonstrated comparatively hydrophobic properties. SLA (Group 3) and modSLA (Group 2) surfaces are made of the same Grade 2 titanium and treated with the same sandblasting and acid‐etching technique (250–500‐μm corundum sandblasting + H_2_SO_4_/HCl acid etching) (Wennerberg, Galli, and Albrektsson 2011). However, they differ because the modSLA (Group 2) surfaces have an additional procedure; laving under nitrogen conservation to avoid air contact and are kept in a sealed glass tube with an isotonic NaCl solution to prevent drying and preserve the clean TiO2 passivation layer, rendering the surface superhydrophilic (Wennerberg, Galli, and Albrektsson 2011). Previous studies have highlighted the benefit of superhydrophilic surfaces' ability to stabilize the initial blood clot and form a well‐organized preliminary collagen‐rich matrix (Schwarz et al. 2007b, 2009). This study further corroborated the enhanced rate of osseointegration of modSLA surfaces compared to SLA surfaces (Buser et al. 2004; Schwarz et al. 2007b; Bornstein et al. 2008; El Chaar et al. 2019). Interestingly and unexpectedly, hydrophilicity in this study did not seem to account for the differences observed in direct bone apposition to the implant surface, as superior results, although not statistically significant, were seen in Group 3 (hydrophobic surface) compared to Group 1 after 8 weeks. Therefore, hydrophilicity is unlikely to explain the difference seen in this study. Nonetheless, after 2 weeks, the results showed that dBIC for Group 2 rather than Group 3 was significantly greater than Group 1, which supports the previous claims that modSLA surface accelerates the process of osseointegration observed by greater BIC compared to SLA surface (Buser et al. 2004; Oates et al. 2007).

Although we cannot exclude surface chemistry as being a factor in the differences seen in this study, previous studies have failed to show a significant difference in osseointegration between these material types. At moderate roughness, both implant materials (cpTi, TiZr) have demonstrated excellent clinical outcomes (Roccuzzo et al. 2014; Karl and Albrektsson 2017; Kowar, Lund, and Stenport 2023). The effects observed in the current study may, therefore, not be related to the differences in implant materials.

In this study, microCT demonstrated no meaningful difference between the geometry of the replica implants (Groups 2 and 3) and the NGA implant (Group 1). However, the characterization of the surfaces with SEM demonstrated differences in micro‐topography and roughness. The NGA implant is reported to be minimally rough (Sa ~0.5 μm), non‐porous at its coronal 2 mm collar, with a nanostructured oxide layer (Milleret et al. 2019). In this study, the relatively smooth neck of the NGA implant at the coronal aspect received GBR. In contrast, Groups 2 and 3 attained the same moderately rough surface along the whole length of the implant. Previous pre‐clinical and clinical studies have demonstrated that moderately rough surfaces (surface roughness, Sa = 1–2 μm) promote osseointegration, whereas smooth (Sa < 0.5 μm) and minimally (Sa = 0.5–1 μm) rough surfaces relatively favor soft tissue attachment (Wennerberg and Albrektsson 2009). Clinical studies investigating subcrestal placement of implants with a smooth (either polished or machined) to rough transition have indicated that such configurations may not yield desirable results for crestal bone maintenance (Hämmerle et al. 1996; Hartman and Cochran 2004). In our study, the surface roughness of the coronal aspect of the NGA measured 0.662 ± 0.176 μm, with the smooth to moderately rough transition occurring at 2 mm from the implant shoulder. Therefore, the differences in fBIC and dBIC with a noticeable absence of bone apposition can be attributed to the NGA implant's smooth 2 mm coronal zone. Additionally, moderately rough surfaces, compared to smooth machined surfaces, demonstrate more extensive blood clot adhesion (Di Iorio et al. 2005), and would explain the differences and the superior results seen for the moderately rough sandblasted acid‐etched implants (Groups 2 and 3). This finding raises an important question: Should the coronal 2 mm of the NGA implant be placed supra or subcrestal in the alveolar ridge and allow soft tissue integration with the smooth 2 mm?

Furthermore, increased porosity has been shown to have a positive effect on osteogenic differentiation and facilitate increased bone ingrowth (Rho, Kuhn‐Spearing, and Zioupos 1998; Vasconcellos et al. 2010; Li, Wang, and Lu 2013). Yet, no consensus on optimal pore size has been achieved (Yao et al. 2021). Group 1 has been reported to transition from a low‐to‐high pore density at its apex (Milleret et al. 2019; Susin et al. 2019). SEM analysis of Groups 2 and 3 demonstrated comparatively higher porosity than the coronal aspect of the NGA (Group 1) surface, and this may also have contributed to the differences observed in this study.

The dehiscence defects were created in as standardized a manner as possible. Anatomical variation between animals was mitigated by pre‐allocating groups equally across anatomical positions, performing appropriate regression analysis with the animal as a factor, and performing the study with a statistically acceptable number of animals.

Finally, it should be noted that the results of this study were obtained through a porcine animal model. The minipig model has been demonstrated to be an appropriate in vivo model to study the osseointegrative process and alveolar remodeling having similar anatomical and healing characteristics to human bone (Musskopf et al. 2022). This study demonstrated osseointegration and alveolar bone remodeling identical to that observed in humans and canine models (Schwarz, Sager, et al. 2010; Schwarz, Jung, et al. 2010; Buser et al. 2013). Therefore, osseointegration in humans may develop similarly in a clinical setting. Nevertheless, clinical studies would be valuable to explore further the differences reported in this study.

## 5 Conclusions

Surface characteristics did not significantly influence the extent of new bone height or bone volume by guided bone regeneration in the defect area. De novo crestal bone formation appears to be primarily influenced by implant surface characteristics, with moderately rough sandblasted acid‐etched surfaces demonstrating significantly higher levels of bone‐to‐implant contact and coronal bone apposition compared to the novel gradient anodized surface.

## Author Contributions

**Shakeel Shahdad:** conceptualization, methodology, funding acquisition, writing – review and editing, data curation, supervision, resources, investigation, validation. **Simon Rawlinson:** writing – review and editing, formal analysis, data curation, supervision. **Nahal Razaghi:** writing – review and editing, data curation, investigation, resources. **Anuya Patankar:** investigation, data curation, resources, writing – review and editing. **Mital Patel:** writing – review and editing, resources, data curation, investigation. **Mario Roccuzzo:** supervision, writing – review and editing, conceptualization, investigation, funding acquisition, methodology, resources, data curation. **Thomas Gill:** writing – original draft, writing – review and editing, formal analysis, data curation, investigation, resources, validation.

## Ethics Statement

This article does not contain any studies with human participants performed by any of the authors. Ethical standards related to the research of human subjects are not applicable. All applicable international, national, and/or institutional guidelines for the care and use of animals were followed.

## Consent

The authors have nothing to report.

## Conflicts of Interest

The present study was funded by a grant from Institut Straumann AG. Shakeel Shahdad, Mital Patel, and Mario Roccuzzo received speaker honorariums for educational courses from Institut Straumann AG. Thomas Gill, Nahal Razaghi, Anuya Patankar, and Simon Rawlinson declare no conflict of interest.

## Supporting information


**Table S1** Association of histomorphometric outcomes and test groups after 2 weeks of healing adjusted for side and position as derived from multivariable mixed linear regression models. ^§^Adjusted parameters were calculated using the factor animal in the model as a random effect. *p‐values were adjusted for multiple comparisons using the Dunnett‐Hsu method. Ref.: Reference level for the comparison for different values of one individual factor. BATA, ratio of bone area to total area; dBIC, bone‐to‐implant contact in the defect area; fBIC, first bone‐to‐implant contact; NBH, new bone height; VBC, vertical bone creep.
**Table S2** Association of histomorphometric outcomes and test groups after 2 weeks of healing adjusted for side and position as derived from multivariable mixed linear regression models. ^§^Adjusted parameters were calculated using the factor animal in the model as a random effect. *p‐values were adjusted for multiple comparisons using the Dunnett‐Hsu method. Ref.: Reference level for the comparison for different values of one individual factor. BATA, ratio of bone area to total area; dBIC, bone‐to‐implant contact in the defect area; fBIC, first bone‐to‐implant contact; NBH, new bone height; VBC, vertical bone creep.

## Data Availability

The data that support the findings of this study are available from the corresponding author upon reasonable request.
