# Supplementary material for: Osseointegration of Anodized vs. Sandblasted Implant Surfaces in a Guided Bone Regeneration Acute Dehiscence‐Type Defect: An In Vivo Experimental Mandibular Minipig Model
Source: Clin Oral Implants Res. 2024 Oct 10;36(1):127–41. doi: 10.1111/clr.14369 (PMC11701963; doi:10.1111/clr.14369)
Supplement: Supplementary file 1 — Table S1 Association of histomorphometric outcomes and test groups after 2 weeks of healing adjusted for side and position as derived from multivariable mixed linear regression models. §Adjusted parameters were calculated using the factor animal in the model as a random effect. *p‐values were adjusted for multiple comparisons using the Dunnett‐Hsu method. Ref.: Reference level for the comparison for different values of one individual factor. BATA, ratio of bone area to total area; dBIC, bone‐to‐implant contact in the defect area; fBIC, first bone‐to‐implant contact; NBH, new bone height; VBC, vertical bone creep. Table S2 Association of histomorphometric outcomes and test groups after 2 weeks of healing adjusted for side and position as derived from multivariable mixed linear regression models. §Adjusted parameters were calculated using the factor animal in the model as a random effect. *p‐values were adjusted for multiple comparisons using the Dunnett‐Hsu method. Ref.: Reference level for the comparison for different values of one individual factor. BATA, ratio of bone area to total area; dBIC, bone‐to‐implant contact in the defect area; fBIC, first bone‐to‐implant contact; NBH, new bone height; VBC, vertical bone creep. [file CLR-36-127-s001.docx]

| Time point | Outcome | Factor | Value | Regression parameters | | | Adjusted parameters for multiple comparisons^§^ | | | | p-value  for the overall effect of the factor |
| --- | --- | --- | --- | --- | --- | --- | --- | --- | --- | --- | --- |
|  |  |  |  | Estimate | SE | p-value | Adjusted mean | 95% CI for the adjusted mean | p-Value*  Ref. level is **group 1** | p-Value*  Ref. level is **group 2** |  |
| 2 Weeks | NBH [µm] | Intercept | I | 1581.946 | 325.28 | 0.0046 |  |  |  |  |  |
|  |  | Group | 1 | -575.997 | 305.92 | 0.1017 | 1239.25 | 725.0358 - 1753.469 | *Ref.* | 0.8021 | 0.2301 |
|  |  |  | 2 | -405.004 | 319.80 | 0.2459 | 1410.25 | 890.4394 - 1930.052 | 0.8083 | *Ref.* |  |
|  |  |  | 3 | 0.000 |  |  | 1815.25 | 1301.932 - 2328.566 | 0.1740 | 0.3881 |  |
|  |  | Side | Left | 260.399 | 255.19 | 0.3415 | 1618.45 | 1215.976 - 2020.922 |  |  | 0.3415 |
|  |  |  | Right | 0.000 |  |  | 1358.05 | 918.5107 - 1797.588 | 0.3415 | 0.3415 |  |
|  |  | Position | Middle | -10.792 | 319.31 | 0.9740 | 1374.35 | 915.8299 - 1832.878 | 0.9991 | 0.9991 |  |
|  |  |  | Middle P. | 320.102 | 334.12 | 0.3699 | 1705.25 | 1231.351 - 2179.144 | 0.5392 | 0.5392 |  |
|  |  |  | Posterior | 0.000 |  |  | 1385.15 | 775.7354 - 1994.556 |  |  | 0.4897 |
| 2 Weeks | VBC [µm] | Intercept | I | 259.280 | 298.68 | 0.4250 |  |  |  |  |  |
|  |  | Group | 1 | -632.014 | 280.90 | 0.0592 | -137.41 | -609.574 - 334.7553 | *Ref.* | 0.0336 | 0.0433 |
|  |  |  | 2 | 217.182 | 293.64 | 0.4836 | 711.79 | 234.4894 - 1189.084 | 0.0342 | *Ref.* |  |
|  |  |  | 3 | 0.000 |  |  | 494.60 | 23.26605 - 965.9434 | 0.1035 | 0.6912 |  |
|  |  | Side | Left | 133.800 | 234.32 | 0.5858 | 423.23 | 53.66807 - 792.7865 |  |  | 0.5858 |
|  |  |  | Right | 0.000 |  |  | 289.43 | -114.166 - 693.0214 | 0.5858 | 0.5858 |  |
|  |  | Position | Middle | 228.883 | 293.20 | 0.4606 | 416.79 | -4.24168 - 837.8119 | 0.6490 | 0.6490 |  |
|  |  |  | Middle P. | 276.393 | 306.79 | 0.3976 | 464.29 | 29.15270 - 899.4368 | 0.5737 | 0.5737 |  |
|  |  |  | Posterior | 0.000 |  |  | 187.90 | -371.671 - 747.4758 |  |  | 0.6584 |
| 2 Weeks | fBIC buccal: First bone to implant contact [µm] | Intercept | I | -2037.787 | 299.02 | 0.0010 |  |  |  |  |  |
|  |  | Group | 1 | -705.455 | 251.27 | 0.0262 | -2616.59 | -3126.04 - -2107.13 | *Ref.* | 0.0047 | 0.0073 |
|  |  |  | 2 | 421.492 | 263.75 | 0.1541 | -1489.64 | -2004.84 - -974.434 | 0.0048 | *Ref.* |  |
|  |  |  | 3 | 0.000 |  |  | -1911.13 | -2418.45 - -1403.81 | 0.0470 | 0.2525 |  |
|  |  | Side | Left | -98.675 | 214.58 | 0.6596 | -2055.12 | -2497.37 - -1612.87 |  |  | 0.6596 |
|  |  |  | Right | 0.000 |  |  | -1956.45 | -2418.86 - -1494.03 | 0.6596 | 0.6596 |  |
|  |  | Position | Middle | 132.093 | 284.90 | 0.6570 | -2049.69 | -2534.33 - -1565.03 | 0.8444 | 0.8444 |  |
|  |  |  | Middle P. | 395.883 | 320.73 | 0.2569 | -1785.90 | -2298.99 - -1272.79 | 0.3863 | 0.3863 |  |
|  |  |  | Posterior | 0.000 |  |  | -2181.78 | -2804.40 - -1559.15 |  |  | 0.4633 |
| 2 Weeks | dBIC: Bone to implant contact [%] | Intercept | I | 7.635 | 5.1608 | 0.1991 |  |  |  |  |  |
|  |  | Group | 1 | -7.904 | 4.8537 | 0.1474 | 2.0899 | -6.06860 - 10.24835 | *Ref.* | 0.0419 | 0.0635 |
|  |  |  | 2 | 5.998 | 5.0738 | 0.2757 | 15.9923 | 7.745122 - 24.23944 | 0.0427 | *Ref.* |  |
|  |  |  | 3 | 0.000 |  |  | 9.9941 | 1.849906 - 18.13830 | 0.2470 | 0.4302 |  |
|  |  | Side | Left | -2.012 | 4.0488 | 0.6345 | 8.3528 | 1.967236 - 14.73836 |  |  | 0.6345 |
|  |  |  | Right | 0.000 |  |  | 10.3647 | 3.391056 - 17.33835 | 0.6345 | 0.6345 |  |
|  |  | Position | Middle | 5.342 | 5.0662 | 0.3267 | 11.3362 | 4.061282 - 18.61101 | 0.4836 | 0.4836 |  |
|  |  |  | Middle P. | 4.752 | 5.3011 | 0.3998 | 10.7459 | 3.227172 - 18.26470 | 0.5765 | 0.5765 |  |
|  |  |  | Posterior | 0.000 |  |  | 5.9942 | -3.67462 - 15.66298 |  |  | 0.5742 |
| 2 Weeks | BA/TA: Bone area/Total area [%] | Intercept | I | 12.850 | 2.7703 | 0.0056 |  |  |  |  |  |
|  |  | Group | 1 | -3.651 | 2.3375 | 0.1623 | 8.6265 | 3.920786 - 13.33213 | *Ref.* | 0.0819 | 0.1122 |
|  |  |  | 2 | 1.873 | 2.4534 | 0.4700 | 14.1511 | 9.392011 - 18.91025 | 0.0837 | *Ref.* |  |
|  |  |  | 3 | 0.000 |  |  | 12.2776 | 7.591559 - 16.96371 | 0.2709 | 0.6764 |  |
|  |  | Side | Left | -1.126 | 1.9955 | 0.5903 | 11.1222 | 7.048030 - 15.19636 |  |  | 0.5903 |
|  |  |  | Right | 0.000 |  |  | 12.2480 | 7.983628 - 16.51228 | 0.5903 | 0.5903 |  |
|  |  | Position | Middle | 0.794 | 2.6459 | 0.7729 | 12.4884 | 8.017506 - 16.95924 | 0.9294 | 0.9294 |  |
|  |  |  | Middle P. | -0.823 | 2.9737 | 0.7900 | 10.8721 | 6.139088 - 15.60511 | 0.9394 | 0.9394 |  |
|  |  |  | Posterior | 0.000 |  |  | 11.6948 | 5.940303 - 17.44920 |  |  | 0.7952 |

^§^The factor animal was introduced in the model as a random effect. The model was a mixed linear regression model

*p-value adjusted for multiple comparisons using the Dunnett-Hsu method

Ref. = Reference level for the comparison within a factor.

**Supplementary Table S1**: Association of histomorphometric outcomes and test groups after 2 weeks of healing adjusted for side and position as derived from multivariable mixed linear regression models. ^§^Adjusted parameters were calculated using the factor animal in the model as a random effect. *p-values were adjusted for multiple comparisons using the Dunnett-Hsu method. Ref.: Reference level for the comparison for different values of one individual factor. Abbreviations: NBH: new bone height, BATA: ratio of bone area to total area, dBIC: bone to implant contact in the defect area, VBC: vertical bone creep, fBIC: first bone to implant contact,

| Time point | Outcome | Factor | Value | Regression parameters | | | Adjusted parameters for multiple comparisons^§^ | | | | p-value  for the overall effect of the factor |
| --- | --- | --- | --- | --- | --- | --- | --- | --- | --- | --- | --- |
|  |  |  |  | Estimate | SE | p-value | Adjusted mean | 95% CI for the adjusted mean | p-Value*  Ref. level is **group 1** | p-Value*  Ref. level is **group 2** |  |
| 8 Weeks | NBH [µm] | Intercept | I | 2365.813 | 326.61 | 0.0002 |  |  |  |  |  |
|  |  | Group | 1 | -311.496 | 291.82 | 0.3086 | 2021.41 | 1500.304 - 2542.508 | *Ref.* | 0.7713 | 0.5791 |
|  |  |  | 2 | -134.971 | 291.82 | 0.6527 | 2197.93 | 1676.829 - 2719.033 | 0.7713 | *Ref.* |  |
|  |  |  | 3 | 0.000 |  |  | 2332.90 | 1811.923 - 2853.880 | 0.4831 | 0.8591 |  |
|  |  | Side | Left | 15.146 | 244.25 | 0.9517 | 2191.65 | 1738.389 - 2644.916 |  |  | 0.9517 |
|  |  |  | Right | 0.000 |  |  | 2176.51 | 1719.403 - 2633.610 | 0.9517 | 0.9517 |  |
|  |  | Position | Middle | -297.737 | 317.80 | 0.3689 | 1926.83 | 1410.261 - 2443.391 | 0.5544 | 0.5544 |  |
|  |  |  | Middle P. | 176.287 | 336.21 | 0.6105 | 2400.85 | 1850.203 - 2951.497 | 0.8184 | 0.8184 |  |
|  |  |  | Posterior | 0.000 |  |  | 2224.56 | 1645.535 - 2803.591 |  |  | 0.3320 |
| 8 Weeks | VBC [µm] | Intercept | I | 2146.491 | 326.18 | 0.0003 |  |  |  |  |  |
|  |  | Group | 1 | -1158.404 | 314.72 | 0.0036 | 640.60 | 136.5350 - 1144.671 | *Ref.* | 0.0088 | 0.0054 |
|  |  |  | 2 | -59.292 | 314.72 | 0.8540 | 1739.71 | 1235.646 - 2243.782 | 0.0088 | *Ref.* |  |
|  |  |  | 3 | 0.000 |  |  | 1799.01 | 1295.127 - 2302.887 | 0.0067 | 0.9745 |  |
|  |  | Side | Left | -510.459 | 258.49 | 0.0739 | 1137.88 | 720.5493 - 1555.208 |  |  | 0.0739 |
|  |  |  | Right | 0.000 |  |  | 1648.34 | 1226.725 - 2069.949 | 0.0739 | 0.0739 |  |
|  |  | Position | Middle | -513.135 | 325.13 | 0.1428 | 972.23 | 489.6860 - 1454.771 | 0.2354 | 0.2354 |  |
|  |  |  | Middle P. | 236.370 | 337.79 | 0.4986 | 1721.73 | 1207.414 - 2236.051 | 0.7075 | 0.7075 |  |
|  |  |  | Posterior | 0.000 |  |  | 1485.36 | 936.5204 - 2034.205 |  |  | 0.0894 |
| 8 Weeks | fBIC buccal: First bone to implant contact [µm] | Intercept | I | -209.749 | 307.82 | 0.5175 |  |  |  |  |  |
|  |  | Group | 1 | -982.491 | 302.90 | 0.0078 | -1614.67 | -2088.12 - -1141.22 | *Ref.* | 0.0046 | 0.0053 |
|  |  |  | 2 | 189.698 | 302.90 | 0.5439 | -442.49 | -915.936 - 30.96496 | 0.0046 | *Ref.* |  |
|  |  |  | 3 | 0.000 |  |  | -632.18 | -1105.43 - -158.927 | 0.0144 | 0.7618 |  |
|  |  | Side | Left | -543.482 | 247.10 | 0.0501 | -1168.19 | -1554.37 - -782.002 |  |  | 0.0501 |
|  |  |  | Right | 0.000 |  |  | -624.71 | -1014.99 - -234.420 | 0.0501 | 0.0501 |  |
|  |  | Position | Middle | -460.528 | 307.23 | 0.1620 | -1206.28 | -1653.25 - -759.310 | 0.2647 | 0.2647 |  |
|  |  |  | Middle P. | 8.450 | 317.40 | 0.9792 | -737.30 | -1213.05 - -261.555 | 0.9995 | 0.9995 |  |
|  |  |  | Posterior | 0.000 |  |  | -745.75 | -1255.60 - -235.904 |  |  | 0.2356 |
| 8 Weeks | dBIC: Bone to implant contact [%] | Intercept | I | 45.143 | 7.2935 | 0.0004 |  |  |  |  |  |
|  |  | Group | 1 | -31.848 | 7.2202 | 0.0010 | 3.5932 | -7.61240 - 14.79880 | *Ref.* | 0.0001 | 0.0002 |
|  |  |  | 2 | 12.773 | 7.2202 | 0.1046 | 48.2143 | 37.00871 - 59.41991 | 0.0001 | *Ref.* |  |
|  |  |  | 3 | 0.000 |  |  | 35.4416 | 24.24069 - 46.64242 | 0.0020 | 0.1790 |  |
|  |  | Side | Left | -13.047 | 5.8766 | 0.0483 | 22.5593 | 13.46217 - 31.65643 |  |  | 0.0483 |
|  |  |  | Right | 0.000 |  |  | 35.6067 | 26.41216 - 44.80132 | 0.0483 | 0.0483 |  |
|  |  | Position | Middle | -8.964 | 7.2791 | 0.2438 | 23.2962 | 12.76714 - 33.82516 | 0.3852 | 0.3852 |  |
|  |  |  | Middle P. | -0.568 | 7.5066 | 0.9410 | 31.6924 | 20.49154 - 42.89327 | 0.9956 | 0.9956 |  |
|  |  |  | Posterior | 0.000 |  |  | 32.2605 | 20.24084 - 44.28018 |  |  | 0.3910 |
| 8 Weeks | BA/TA: Bone area/Total area [%] | Intercept | I | 46.646 | 4.8157 | <.0001 |  |  |  |  |  |
|  |  | Group | 1 | -6.241 | 4.7673 | 0.2172 | 42.4789 | 35.08011 - 49.87773 | *Ref.* | 0.9372 | 0.4206 |
|  |  |  | 2 | -4.826 | 4.7673 | 0.3332 | 43.8944 | 36.49556 - 51.29319 | 0.9372 | *Ref.* |  |
|  |  |  | 3 | 0.000 |  |  | 48.7200 | 41.32432 - 56.11570 | 0.3533 | 0.5159 |  |
|  |  | Side | Left | -0.985 | 3.8802 | 0.8042 | 44.5385 | 38.53186 - 50.54513 |  |  | 0.8042 |
|  |  |  | Right | 0.000 |  |  | 45.5237 | 39.45273 - 51.59469 | 0.8042 | 0.8042 |  |
|  |  | Position | Middle | 4.042 | 4.8062 | 0.4182 | 46.5069 | 39.55484 - 53.45899 | 0.6153 | 0.6153 |  |
|  |  |  | Middle P. | 3.657 | 4.9564 | 0.4761 | 46.1217 | 38.72604 - 53.51741 | 0.6826 | 0.6826 |  |
|  |  |  | Posterior | 0.000 |  |  | 42.4647 | 34.52835 - 50.40100 |  |  | 0.6753 |

^§^The factor animal was introduced in the model as a random effect. The model was a mixed linear regression model

*p-value adjusted for multiple comparisons using the Dunnett-Hsu method

Ref. = Reference level for the comparison within a factor.

**Supplementary Table S2**: Association of histomorphometric outcomes and test groups after 8 weeks of healing adjusted for side and position as derived from multivariable mixed linear regression models. ^§^Adjusted parameters were calculated using the factor animal in the model as a random effect. *p-values were adjusted for multiple comparisons using the Dunnett-Hsu method. Ref.: Reference level for the comparison for different values of one individual factor. Abbreviations: NBH: new bone height, BATA: ratio of bone area to total area, dBIC: bone to implant contact in the defect area, VBC: vertical bone creep, fBIC: first bone to implant contact.
